# Impact of COVID-19 Pandemic on Oncological Surgery Activities: A Retrospective Study from a Southern Italian Region

**DOI:** 10.3390/healthcare10112329

**Published:** 2022-11-21

**Authors:** Giuseppe Di Martino, Fabrizio Cedrone, Pamela Di Giovanni, Ferdinando Romano, Tommaso Staniscia

**Affiliations:** 1 Department of Medicine and Ageing Sciences, “G. d’Annunzio” University of Chieti-Pescara, Via dei Vestini 31, 66100 Chieti, Italy; 2 Local Health Authority of Pescara, Via Paolini, 65100 Pescara, Italy; 3 Department of Pharmacy, “G. d’Annunzio” University of Chieti-Pescara, Via dei Vestini 31, 66100 Chieti, Italy; 4 Department of Infectious Diseases and Public Health, “La Sapienza” University of Rome, Piazza Aldo Moro 5, 00138 Rome, Italy

**Keywords:** cancer, COVID-19, surgery, breast cancer, colorectal cancer, HDR, Italy

## Abstract

(1) Background: The pandemic had a strong impact on healthcare for other diseases, the so-called collateral damage. This situation heavily impacted the health care system, causing a deferment of surgical admissions. This situation had an immediate and long-term impact on millions of patients with surgical diseases all over the world. The objective of this study was to evaluate the incidence of hospitalizations for colorectal and breast cancers in an Italian region in the year 2020 and compare it with the years 2018–2019. (2) Methods: This retrospective study was performed in the region of Abruzzo, Italy. Monthly number of hospitalizations in the year 2020 was compared with a control period consisting of the average of admissions that occurred in the years 2018–2019 using Poisson regression. (3) Results: A reduction in hospital admissions for all diseases considered was found. In particular, compared with years 2018–2019, admissions for colorectal cancer were 35.71% lower (HRR 0.915; *p* < 0.001), and admissions for breast cancer were 10.36% lower (HRR 0.895; *p* < 0.001) (4) Conclusions: The results of this study showed the decrease of admissions for elective oncological surgery during pandemic, suggesting the need of strategic measures to face the burden of future years’ hospitalizations.

## 1. Introduction

From December 2019, an emerging severe acute respiratory syndrome caused by a novel coronavirus 2 (COVID-19) was identified in China [1]. During the first four months of 2020, it rapidly spread around the globe [2], and the World Health Organization (WHO) classified it as pandemic [3]. From the beginning of the epidemic, Italy has been one of the most-affected countries, and the Italian government imposed a national lockdown on 9 March 2020, in order to mitigate the raising infection rate [4]. The pandemic heavily impacted health systems with high hospitalization for COVID-19, forcing the transformation of a great proportion of Italian hospitals into COVID-19 centers [4]. This situation, which helped healthcare services to combat the pandemic had, on the other hand, indirect consequences on the care for other diseases, the so-called collateral damage [5,6]. Routine diagnostic procedures and elective hospitalizations were cancelled or deferred in order to focus the great part of resources on the care of COVID-19 patients [7]. This could be useful for two main reasons: firstly, it shielded patients from hospital sars-cov-2 transmission; secondly, it spared personal protective equipment to be saved for COVID-19 care and released ward and intensive care beds for peak capacity for such patients [8]. This situation had immediate and long-term impact on millions of surgical patients all over the world. Globally, there was shortfall in surgical service delivery and recovery from the COVID-19 pandemic [9]. Postponing elective oncological surgery will have a devastating impact on healthcare systems: delaying time-sensitive elective operations, such as critical oncological admissions, may lead to worsening outcomes and preventable deaths [10,11].

The Abruzzo region during 2019 registered 1079 new cases of colorectal cancer and 1101 new cases of breast cancer [12]. Evaluating the impact of the first year of the pandemic on the surgical activities for the most common oncological diseases [12], such as breast and colorectal cancer, will provide possible baseline data to plan the post-pandemic surgical recovery. 

This study aimed to evaluate the incidence of admissions for colorectal and breast cancer during year 2020 in an Italian southern region and compare it with to 2018–2019 years.

## 2. Materials and Methods

### 2.1. Study Design and Setting

This retrospective study was performed in Abruzzo, a region in southern Italy. Abruzzo’s population counts more than 1.2 million inhabitants and its healthcare service is organized into four different local health authorities (LHA). Each LHA has a referral tertiary hospital [13]. All tertiary hospitals have a general surgery ward where major oncological surgeries are performed. Regarding breast cancer, the Abruzzo region focused the surgery activity on two main hub centers (Ortona and L’Aquila Hospitals), and secondly in two spokes (Pescara and Teramo Hospital). As indicated by Italian Ministry of Health, Abruzzo organized three oncological screening campaigns:

- Breast cancer: mammography for all women aged between 50 and 69 years;
- Colon cancer: stool blood occult test for all inhabitants aged between 50 and 70 years;
- Cervical cancer: Pap test or HPV-DNA test for all women aged between 25 and 64 years of age.

All LHAs in Abruzzo actively invited all patients involved in the screening campaigns.

### 2.2. Data Collection

Data were collected from hospital discharge records (HDR), which report data on patients’ demographics and diagnosis-related groups (DRG) used to categorize the admission. Each HDR record, in addition, reports a maximum of 6 diagnoses (1 principal diagnosis and up to 5 comorbidities) and up to 6 procedures performed during the admission. Diagnoses and procedures were coded accordingly to the International Classification of Disease, 9th Revision, Clinical Modification (ICD-9-CM, National Center for Health Statistics (NCHS) and the Centers for Medicare and Medicaid Services External, Atlanta, GA, USA). The study considered hospitalizations according to the following categories coded as defined here:

- Malignant Colon Cancer: all non-emergency admissions discharged with ICD-9-CM codes: 153.x, 197.5 (Colon Cancer) as principal or secondary diagnosis and total or partial colectomy as principal or secondary intervention (ICD-9-CM: 45.7x, 45.8, 45.9x, 46.03, 46.04, 46.1x). Admissions with rectal resection as principal or secondary intervention (ICD-9-CM: 48.49, 48.5, 48.6) were excluded;
- Malignant Rectal Cancer: all non-emergency admissions discharged with ICD-9-CM codes: 154.x, 197.5 (Malignant Rectal Cancer) and rectal resection (ICD-9-CM: 48.49, 48.5, 48.6x) as principal or secondary intervention. Admissions with total or partial colon resection as principal or secondary intervention (ICD-9-CM: 45.7x, 45.8, 45.9x, 46.03, 46.04, 46.1x) were excluded;
- Malignant Breast Cancer: all non-emergency admissions discharged with ICD-9-CM codes: 174.x, 198.81, 233.0 (Malignant Breast Cancer) and total or partial mastectomy (ICD-9-CM: 85.2x, 85.33, 85.34, 85.35, 85.36, 85.4x) as principal or secondary intervention. Only admissions that occurred among females were included.

In addition, hospital length of stay (LOS) and in-hospital death were extracted from HDR. 

### 2.3. Statistical Analysis

Continuous variables were described as mean ± standard deviation (SD) or median and interquartile range (IQR) according to the distribution of data. Categorical variables were described as frequency and percentage. Qualitative variables were compared with Pearson’s Chi-Squared test or Fisher’s exact test as appropriate. Continuous variables were compared with Mann–Whitney U Test. The number of hospitalizations that occurred in 2020 for each cancer was compared with the average of the admissions that occurred during the previous two years (2018–2019). The average of admissions that occurred in years 2018–2019 was calculated as the arithmetic mean of cause-specific hospitalizations that occurred in each considered year. In addition, the monthly number of hospitalizations in the year 2020 was reported in parallel with the average of the monthly number of admissions that occurred in 2018–2019. Incidence rate ratios comparing the year 2020 with the control periods was reported as Hospitalization Rate Ratio (HRR) using a 95% confidence interval (95% CI). HRR was calculated by constructing a Poisson regression model for each disease, adjusted for age, gender, and hospital, considering the calendar year as the independent variable. The statistical significance in all analyses was set at a *p*-value ≤ 0.05. Statistical analyses were performed using STATA v14 software (Stata Corp LLC, College Station, TX, USA).

## 3. Results

In the Abruzzo region, during the year 2020, a total of 604 patients were admitted for colorectal cancer surgery, and 1012 patients were admitted for breast cancer surgery, as reported in Table S1. Analyzing only the pandemic period, between March 2020 and December 2020, a total of 479 patients were admitted for colorectal cancer surgery, and 815 patients were admitted for breast cancer surgery, as reported in Table 1.

Significant difference among age class for both considered diseases were observed. In particular, a reduction in the age class 18–44 was observed for colorectal cancer (−30%) and for breast cancer (−38.5%). No differences in LOS were observed for either disease during the study periods. A global reduction in hospital admissions for both surgeries was observed, as reported in Table S2. In particular, compared with the previous two years, in 2020, admissions for colorectal cancer were 44.5 lower (−35.71%; HRR 0.915; 95% CI: 0.914–0.916; *p* < 0.001), for breast cancer were 66.5 lower (−10.36%; HRR 0.895; 95% CI: 0.894–0.897; *p* < 0.001). Analyzing only the pandemic period (from March 2020 to December 202), similar results were obtained: HRR 0.879 (95% CI: 0.877–0.880; *p* < 0.001) for colorectal cancer and HRR 0.862 (95% CI: 0.861–0.864; *p* < 0.001) for breast cancer (Table 2).

**Figure 1:**
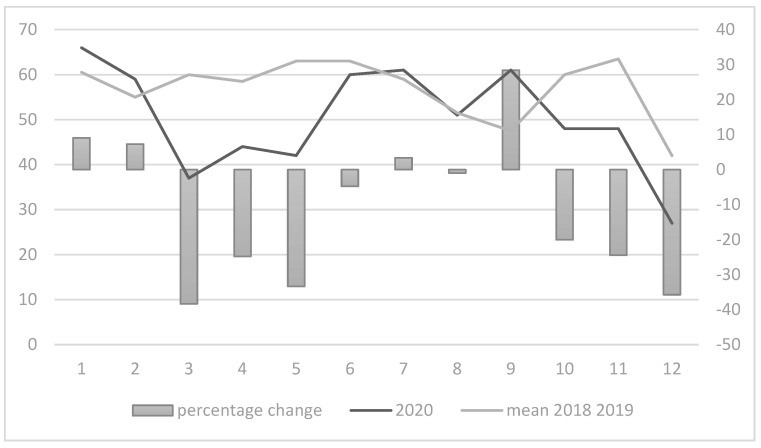
Monthly differences in colorectal cancer admissions.

**Figure 2:**
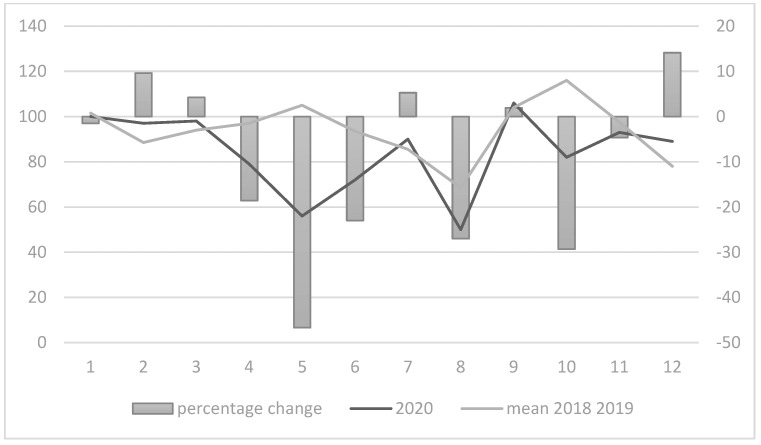
Monthly differences in breast cancer admissions.

Considering the monthly incidence of hospitalization, a significant reduction was observed between March and June 2020, and during the last two months of the year, for both colorectal and breast cancers, as shown in Figure 1 and Figure 2. In particular, during the period between March and May, a strong reduction was observed in colorectal and breast cancer admissions (more than 40% less for both colorectal in March 2020 and breast cancer admissions in May 2020). A turnaround in admissions for colorectal cancer was observed during July and September, with a second decrease in admissions between October and December 2020. Regarding breast cancer, a strong increase in admissions was observed only in December (+12.3%). 

## 4. Discussion

The COVID-19 pandemic had a heavy impact on healthcare services worldwide. This study reported the lowering in hospital admission rates for colorectal and breast cancer surgery that occurred during 2020 in a southern Italian region, compared with the previous two years as the control period. During the year 2020, there was a significant decrease in hospitalization for colorectal and breast cancer surgeries compared with the previous two years. These results are in line with the previous literature, confirming findings reported across the world [9,14]. Recent literature highlighted a decreasing trend in surgical activities, in particular for colorectal and hepatic cancers [14,15,16]. The national lockdown clearly affected the surgical activity, with a low admission rate during first four months of the year 2020 for elective oncological surgery. The observed reduction in oncological surgery can be explained by the transformation of the healthcare system to fit the needs of the pandemic. The conversion of a great proportions of surgical units into COVID wards and the decrease in the availability of intensive care unit (ICU) beds and surgical rooms strongly affected the routine surgical activity [16]. As consequence, oncological patients whose surgery was postponed could experience an advancement of the disease with worse oncological staging at the treatment [14,17]. In addition, it can cause worse surgical outcomes, as previously reported [14]. The lower decrease in breast cancer admissions compared with colorectal cancer can be explained by an important point: in the Abruzzo region [18], breast cancer surgery units were organized in two hub hospitals, Ortona Hospital and L’Aquila Hospital. These units are specialized in this type of surgery and their activity is focused mainly on the treatment of this disease. On the other hand, colorectal surgery, due to the larger incidence of the diseases impacting both genders, was performed in a great many of the general surgery units of the Abruzzo region. General surgery units, as priorly stated, were involved in the ward conversion in order to assist COVID-19 patients. The restart of the normal surgical activity and the reconversion of surgical wards helped to improve the surgical activity, with a strong increase in surgeries, particularly in September 2020 (Tables S3 and S4).

In parallel, the lockdown also influenced cancer-screening activities which were suspended between March and June 2020. The lack in cancer screening also had a strong impact on cancer surgery, causing a decrease of new cancer diagnoses, particularly among asymptomatic patients. This point is in line with other countries that experienced a decrease in oncological surgery in parallel with the decrease in cancer screenings [19,20,21]. The sustained lockdown cannot reduce the performance of the screenings, but it could negatively affect the stage and the mortality rate of future cancer diagnoses. In addition, the reactivation of screening activities was not immediate, and it was unequal across Italian regions [14,22,23]. This situation can be translated into a lack of oncological diagnoses that will cause an increase in late-stage diagnoses during the coming years [21]. Both these situations will cause an overload in surgical waiting lists [14,15,16,17], with the need for a larger availability in surgical sessions [21].

Regarding differences among age groups, this study showed a significant decrease among all age classes, in particular among younger patients (age 18–44), for both cancers. Despite the fact that, among the 0–44-year-old population, breast cancer is the leading cause of cancer diagnosis among women [24], the pandemic and the block in screening programs, likely led to an increase in symptomatic cancer admissions that typically affect older patients, compared with asymptomatic cancer diagnosed by screening. The same observation can be made for colorectal cancer, which represents the most frequent cancer, impacting both genders [24] (Table S3). 

### Strengths and Limitations

The strength of this study is the homogenous and wide sample analyzed and the long study period considered. This is the first study conducted in Italy reporting data on surgical activities and admissions that occurred in an entire region, making these data generalizable. In the other hand, this study has several limitations. First, the selection of diagnosis based on ICD-9-CM codes was not able to evaluate the severity of the studied conditions. Second, the use of HDR was lacking in some clinical information, such as drug therapy or tumor stage, and some diagnosis codes could be under-reported or miscoded. Third, the retrospective nature of the study did not allow for the evaluation of the prospective impact of pandemic of surgery organization, which was different by LHA and single hospital. In addition, the real incidence of these types of cancers cannot be estimated from HDR.

## 5. Conclusions

The results of this study are relevant for public health authorities and surgery physicians. They showed the impact of the COVID-19 pandemic on surgery volume for two common oncological surgeries. These results highlighted the necessity of strategic measures to deal with new cancer diagnoses and surgery overload during the coming years. In addition, these results can help policymakers to develop strategic measures to face future event lock-downs. All LHAs should organize and prioritize their activities in order to treat both prior and new cancer diagnoses. This necessity will require the improvement in healthcare workers, bed availability, and increasing routine diagnostic capacity.

## Figures and Tables

**Table 1:** Comparison of admissions that occurred between the last ten months of 2020 (March-December) and the mean of the years 2018–2019.

	2018–2019 Mean (SD)	2020 N	Diff (%)	*p*-Value
**Colorectal Cancer**				
Admissions	538 (73.5)	479	−8.9	
Age				
18–44	40 (7.4)	10 (2.1)	−25	<0.001 *
45–74	281.5 (52.3)	255 (53.2)	−9.4	
≥75	216.5 (40.2)	214 (44.7)	−1.2	
LOS median (IQR)	11 (8–17)	10 (7–16)		0.184 ^+^
In-hospital Deaths N(%)	21.5 (4.0)	20 (4.17)	−9.3	0.245 *
**Breast Cancer**				
Admissions	894 (86.2)	815	−9.1	
Age				
18–44	135.5 (15.2)	97 (11.9)	−28.5	<0.001 *
45–74	542.5 (60.7)	543 (66.6)	0.1	
≥75	216 (24.2)	175 (21.5)	−18.9	
LOS median (IQR)	2 (1–3)	2 (1–3)		0.189 ^+^
In-hospital Deaths N(%)	0 (0.00)	0 (0.00)		NA

* Pearson’s Chi-Squared Test. ^+^ Mann–Whitney U test. LOS: length of stay; IQR: interquartile range; NA: Not applicable.

**Table 2:** Hospitalization rate ratios of colorectal and breast cancer in last ten months of 2020 compared with the two-year period 2018–2019.

	HRR (95% CI)	*p*-Value *
**Colorectal cancer**		
2020 vs. 2018–2019	0.879 (0.877–0.880)	<0.001
**Breast cancer**		
2020 vs. 2018–2019	0.862 (0.861–0.864)	<0.001

* All models were adjusted for hospital, age, and gender (only for colorectal cancer). HRR: hospitalization rate ratio; 95% CI: 95% confidence interval.

## Data Availability

The data presented in this study are available on request from the corresponding author. The data are not publicly available due to privacy and ethical reasons.

## Supplementary Materials

The following supporting information can be downloaded at: https://www.mdpi.com/article/10.3390/healthcare10112329/s1, Table S1: Comparison of admissions occurred between 2020 and the mean of the years 2018–2019; Table S2: Hospitalization rate ratios of colorectal and breast cancer in 2020 compared to the two-year period 2018–2019; Table S3: Admissions for Colorectal cancer surgery by hospital; Table S4: Admissions for Breast cancer surgery by hospital.
