# Supplementary material for: Impact of COVID-19 Pandemic on Oncological Surgery Activities: A Retrospective Study from a Southern Italian Region
Source: Healthcare (Basel). 2022 Nov 21;10(11):2329. doi: 10.3390/healthcare10112329 (PMC9691147; doi:10.3390/healthcare10112329)
Supplement: Supplementary file 1 [file healthcare-10-02329-s001.zip › healthcare-1911438-SI.pdf]

Supplementary materials

Table S1. Comparison of admissions occurred between 2020 and the mean of the years 2018-19

	**2018-2019** *mean (SD)*	**2020** *N*	**Diff.** **%**	**p-value**
**Colorectal Cancer**				
Admissions	648.5 (±85.56)	604	-35.71	
Age				
18-44	52 (8.02)	13 (2.15)	-75.00	
45-74	331 (51.04)	327 (54.14)	-1.20	<0.001*
≥75	265.5 (40.94)	264 (43.71)	-0.56	
LOS median (IQR)	10 (7-16)	10 (7-15)		0.197⁺
In-hospital Death N(%)	25.5 (3.93)	22 (3.64)	-13.72	0.180*
**Breast Cancer**				
Admissions	1078.5 (±82.73)	1012	-10.36	
Age				
18-44	168 (15.58)	114 (11.26)	-32.14	
45-74	649.5 (60.22)	676 (66.80)	+4.08	<0.001*
≥75	261 (24.20)	222 (21.94)	-14.94	
LOS median (IQR)	2 (1-3)	2 (1-3)		0.189
In-hospital Death N(%)	0 (0.00)	0 (0.00)		NA

*Pearson's Chi-Squared Test

⁺Mann-Whitney U test

LOS: length of stay; IQR: interquartile range; NA: Not applicable

Table S2. Hospitalization rate ratios of colorectal and breast cancer in 2020 compared to the two-year period 2018-19

	**HRR (95%CI)**	**p-value***
	**Colorectal cancer**	
2020 vs. 2018-2019	0.915 (0.914-0.916)	<0.001
	**Breast cancer**	
2020 vs. 2018-2019	0.895 (0.894-0.897)	<0.001

*All models were adjusted for hospital, age and gender (only for colorectal cancer)

HRR: hospitalization rate ratio; 95%CI: 95% confidence interval;

Table S3. Admissions for Colorectal cancer surgery by hospital

		**Age Class**		
**Hospitals**		18-44	45-74	>=75
L'Aquila Hospital	2018	0	30	23
	2019	10	30	17
	2020	1	33	12
Sulmona Hospital	2018	0	6	5
	2019	2	14	4
	2020	0	7	10
Avezzano Hospital	2018	1	26	22
	2019	9	35	18

	2020	1	21	19
Castel di Sangro Hospital	2018	0	0	0
	2019	1	1	1
	2020	0	0	0
Tagliacozzo Hospital	2018	0	0	0
	2019	0	1	0
	2020	0	0	0
Private Hospital 1	2018	0	5	9
	2019	0	6	8
	2020	0	8	0
Private Hospital 2	2018	0	0	0
	2019	0	2	0
	2020	0	0	0
Teramo Hospital	2018	1	11	18
	2019	10	26	20
	2020	1	38	27
Giulianova Hospital	2018	0	10	6
	2019	1	15	10
	2020	0	2	8
Atri Hospital	2018	1	15	13
	2019	2	10	9
	2020	0	12	10
S Omero Hospital	2018	0	10	8
	2019	5	9	8
	2020	1	8	14
Pescara Hospital	2018	3	59	61
	2019	11	33	17
	2020	4	43	25
Penne Hospital	2018	0	15	11
	2019	2	2	5
	2020	1	9	7
Popoli Hospital	2018	0	2	1
	2019	1	2	1
	2020	0	9	3
Private Hospital 3	2018	1	21	23
	2019	2	16	11
	2020	0	32	30
Private Hospital 4	2018	1	53	38
	2019	1	22	16
	2020	2	48	40
Chieti Hospital	2018	3	48	35
	2019	14	22	15
	2020	0	16	20
Lanciano Hospital	2018	0	10	20
	2019	7	8	10
	2020	1	17	11

Vasto Hospital	2018	2	32	17
	2019	9	13	16
	2020	1	11	12
Atessa Hospital	2018	0	0	0
	2019	0	0	3
	2020	0	0	0
Ortona Hospital	2018	0	6	2
	2019	1	7	8
	2020	0	0	0
Private Hospital 5	2018	0	0	0
	2019	0	3	2
	2020	0	0	0
Private Hospital 6	2018	0	0	0
	2019	1	3	1
	2020	0	0	0
Private Hospital 7	2018	0	9	4
	2019	1	8	3
	2020	0	5	9
Private Hospital 8	2018	0	0	0
	2019	0	0	2
	2020	0	0	0

Table S4. Admissions for Breast cancer surgery by hospital

		**Age Class**		
**Hospitals**		18-44	45-74	>=75
L'Aquila Hospital	2018	0	30	23
	2019	10	30	17
	2020	1	33	12
Sulmona Hospital	2018	0	6	5
	2019	2	14	4
	2020	0	7	10
Avezzano Hospital	2018	1	26	22
	2019	9	35	18
	2020	1	21	19
Castel di Sangro Hospital	2018	0	0	0
	2019	1	1	1
	2020	0	0	0
Tagliacozzo Hospital	2018	0	0	0
	2019	0	1	0
	2020	0	0	0
Private Hospital 1	2018	0	5	9
	2019	0	6	8
	2020	0	8	0
Private Hospital 2	2018	0	0	0

	2019	0	2	0
	2020	0	0	0
Teramo Hospital	2018	1	11	18
	2019	10	26	20
	2020	1	38	27
Giulianova Hospital	2018	0	10	6
	2019	1	15	10
	2020	0	2	8
Atri Hospital	2018	1	15	13
	2019	2	10	9
	2020	0	12	10
S Omero Hospital	2018	0	10	8
	2019	5	9	8
	2020	1	8	14
Pescara Hospital	2018	3	59	61
	2019	11	33	17
	2020	4	43	25
Penne Hospital	2018	0	15	11
	2019	2	2	5
	2020	1	9	7
Popoli Hospital	2018	0	2	1
	2019	1	2	1
	2020	0	9	3
Private Hospital 3	2018	1	21	23
	2019	2	16	11
	2020	0	32	30
Private Hospital 4	2018	1	53	38
	2019	1	22	16
	2020	2	48	40
Chieti Hospital	2018	3	48	35
	2019	14	22	15
	2020	0	16	20
Lanciano Hospital	2018	0	10	20
	2019	7	8	10
	2020	1	17	11
Vasto Hospital	2018	2	32	17
	2019	9	13	16
	2020	1	11	12
Atessa Hospital	2018	0	0	0
	2019	0	0	3
	2020	0	0	0
Ortona Hospital	2018	0	6	2
	2019	1	7	8
	2020	0	0	0
Private Hospital 5	2018	0	0	0
	2019	0	3	2
	2020	0	0	0

Private Hospital 6	2018	0	0	0
	2019	1	3	1
	2020	0	0	0
Private Hospital 7	2018	0	9	4
	2019	1	8	3
	2020	0	5	9
Private Hospital 8	2018	0	0	0
	2019	0	0	2
	2020	0	0	0
